# Automated segmentation of lesions and organs at risk on [^68^Ga]Ga-PSMA-11 PET/CT images using self-supervised learning with Swin UNETR

**DOI:** 10.1186/s40644-024-00675-x

**Published:** 2024-02-29

**Authors:** Elmira Yazdani, Najme Karamzadeh-Ziarati, Seyyed Saeid Cheshmi, Mahdi Sadeghi, Parham Geramifar, Habibeh Vosoughi, Mahmood Kazemi Jahromi, Saeed Reza Kheradpisheh

**Affiliations:** 1 Medical Physics Department, School of Medicine, Iran University of Medical Sciences, Tehran, 14155-6183, Iran; 2 Fintech in Medicine Research Center, Iran University of Medical Sciences, Tehran, Iran; 3 Research Center for Nuclear Medicine, Tehran University of Medical Sciences, Tehran, Iran; 4 Department of Computer and Data Sciences, Faculty of Mathematical Sciences, Shahid Beheshti University, Tehran, Iran; 5 Nuclear Medicine and Molecular Imaging Department, Imam Reza International University, Razavi Hospital, Mashhad, Iran

**Keywords:** Prostate cancer, PSMA, PET/CT, Segmentation, Lesion detection, Neural network, Deep-learning, Swin UNETR, Self-supervised learning

## Abstract

**Background:**

Prostate-specific membrane antigen (PSMA) PET/CT imaging is widely used for quantitative image analysis, especially in radioligand therapy (RLT) for metastatic castration-resistant prostate cancer (mCRPC). Unknown features influencing PSMA biodistribution can be explored by analyzing segmented organs at risk (OAR) and lesions. Manual segmentation is time-consuming and labor-intensive, so automated segmentation methods are desirable. Training deep-learning segmentation models is challenging due to the scarcity of high-quality annotated images. Addressing this, we developed shifted windows UNEt TRansformers (Swin UNETR) for fully automated segmentation. Within a self-supervised framework, the model’s encoder was pre-trained on unlabeled data. The entire model was fine-tuned, including its decoder, using labeled data.

**Methods:**

In this work, 752 whole-body [^68^Ga]Ga-PSMA-11 PET/CT images were collected from two centers. For self-supervised model pre-training, 652 unlabeled images were employed. The remaining 100 images were manually labeled for supervised training. In the supervised training phase, 5-fold cross-validation was used with 64 images for model training and 16 for validation, from one center. For testing, 20 hold-out images, evenly distributed between two centers, were used. Image segmentation and quantification metrics were evaluated on the test set compared to the ground-truth segmentation conducted by a nuclear medicine physician.

**Results:**

The model generates high-quality OARs and lesion segmentation in lesion-positive cases, including mCRPC. The results show that self-supervised pre-training significantly improved the average dice similarity coefficient (DSC) for all classes by about 3%. Compared to nnU-Net, a well-established model in medical image segmentation, our approach outperformed with a 5% higher DSC. This improvement was attributed to our model’s combined use of self-supervised pre-training and supervised fine-tuning, specifically when applied to PET/CT input. Our best model had the lowest DSC for lesions at 0.68 and the highest for liver at 0.95.

**Conclusions:**

We developed a state-of-the-art neural network using self-supervised pre-training on whole-body [^68^Ga]Ga-PSMA-11 PET/CT images, followed by fine-tuning on a limited set of annotated images. The model generates high-quality OARs and lesion segmentation for PSMA image analysis. The generalizable model holds potential for various clinical applications, including enhanced RLT and patient-specific internal dosimetry.

**Supplementary Information:**

The online version contains supplementary material available at 10.1186/s40644-024-00675-x.

## Background

Prostate cancer (PC) is the most prevalent malignancy in men and ranks as the third leading cause of cancer-related deaths among men worldwide [1, 2]. The limited efficacy of advanced treatment options often leads to disease progression in many cases of PC toward metastatic castration-resistant prostate cancer (mCRPC) [3–5]. A multidisciplinary approach should be used at this stage, including chemotherapy, external beam radiotherapy, radioligand therapy (RLT), and hormonal therapies [6–8].

Prostate-specific membrane antigen (PSMA) is a transmembrane glycoprotein, physiologically expressed in several tissues [9, 10]. A high expression of PSMA is observed on the surface of most primary prostate cancer cells and metastatic lesions [11, 12]. PSMA is an effective target for PC imaging and therapy, such as its labeling with ^68^Ga and ^177^Lu as theranostics pair [2, 7, 13–15]. PSMA-focused positron emission tomography/ computed tomography (PET/CT) imaging, especially with [^68^Ga]Ga-PSMA-11, has become the gold standard in PC diagnosis [16]. Because of its unparalleled sensitivity and specificity in lesion detection, it enables precise identification of metastatic sites [17, 18]. PSMA PET/CT shows promise for various purposes, such as early recurrence detection, prognosis, (re)staging, treatment planning, treatment follow-up, response rate, and dose prediction [19, 20]. However, its practical use in these applications is limited by labor-intensive and error-prone manual segmentation.

Deep learning (DL) models are increasingly used for semi-automated and automated segmentation in PSMA PET/CT [19–24]. However, accurate segmentation remains a significant challenge due to various factors. These include noise, motion artifacts, and differences in the location, texture, shape, and appearance of tumors among patients [25]. Developing a precise tumor segmentation model that minimizes false positive annotations in PET/CT image analysis is crucial, especially for scans with widespread lesion metastases like [^68^Ga]Ga-PSMA-11 PET/CT of mCRPC cases. Meanwhile, the initial segmentation of normal organs with consistent spatial characteristics can pave the way for automated tumor segmentation.

Over the past decade, various artificial intelligence (AI) techniques in medical imaging have emerged [26, 27]. Many studies have focused on supervised learning algorithms and convolutional neural networks (CNNs) in medical image segmentation, particularly PET or PET/CT segmentation [24]. Despite their growing popularity, these methods have limitations; their limited receptive field is one of their drawbacks. Introducing the vision transformer (ViT) revolutionized computer vision with its capability to learn global and local information [28]. ViTs employ self-attention blocks that process a more comprehensive range of image patches, encoding visual representations as sequences. This approach allows them to model global information more effectively, overcoming some limitations inherent in CNNs [28].

Traditional algorithms highly depend on abundant, accurately labeled data, while gathering and accurately annotating data for medical tasks is challenging for such algorithms. To address this issue, a novel learning paradigm known as self-supervised learning (SSL) has emerged [29]. SSL is a type of unsupervised learning that does not need explicit labels for the data. Instead, it generates pseudo-labels from the data using pretext tasks, such as masking, predicting, or reconstructing parts of the data [30]. The models can learn meaningful and generalizable data representations by solving these pretext tasks, which can then be used for downstream tasks, such as segmentation or classification. SSL offers a promising alternative to traditional algorithms, as it can leverage the abundant unlabeled data in the medical domain. This approach can reduce the dependence on human annotations, where annotating and masking such data is challenging, as it requires expert knowledge, consensus, and privacy protection [31].

In the present work, we performed and evaluated the shifted windows UNEt TRansformers (Swin UNETR), a hierarchical ViT, for a fully automated patient segmentation of [^68^Ga]Ga-PSMA-11 PET/CT images. This 3D transformer-based model has previously shown remarkable performance in 3D semantic segmentation tasks in various medical modalities, including magnetic resonance imaging and computerized tomography (CT) scans [32, 33]. After extensive experimentation, which involved exploring various aspects of network design, we successfully implemented a configuration that consistently yielded superior results for 3D volume PET/CT images. This configuration effectively captures contextual information from neighboring regions while maintaining computational efficiency and preserving the global context. In this regard, we initially pre-trained the model’s encoder on unlabeled data within a self-supervised framework. This framework involved three proxy tasks: rotation prediction, inpainting, and contrastive coding. Subsequently, the entire model was fine-tuned, including its decoder, using labeled data annotated meticulously by experts.

Our code is available at: https://github.com/ElmiraYazdani/Lesions-OARs-Segmentation-PSMA-PETCT-SSL-SwinUNETR.

## Materials and methods

### Data description

The inclusion criteria for the retrospective study comprised cases with a [^68^Ga]Ga-PSMA-11 PET/CT scan conducted between January 2021 and June 2023. The exclusion criteria were those with insufficient image quality, low dose, or those cases where PET or CT scan data were absent from the database. Patients underwent [^68^Ga]Ga-PSMA-11 PET/CT scans for clinical purposes, including (re)staging or treatment response assessment. A low-dose CT non-contrast image was acquired to correct attenuation and determine anatomical location. The study was conducted according to local research committee requirements and ethical guidelines.

The production of ^68^Ga used a standard ^68^Ge/^68^Ga generator, and the labeling of PSMA-HBED-CC with ^68^Ga was performed. The dataset included 752 PET/CT images from two nuclear medicine centers, i.e., center A (Siemens BioGraph6 TruePoint TrueV, 408 patients) and center B (Siemens BioGraph6 TruePoint, 344 patients) following the intravenous injection of 150–220 MBq [^68^Ga]Ga-PSMA-HBED-CC.

Prior to the study, quality control of the protocol at each site revealed no evidence of bias [34]. A 3-D acquisition mode was used for all PE/CT images following a 45 to 60-minute uptake period from the skull to the mid-thigh. Subsequently, decay and scatter correction were applied, and images underwent iterative reconstruction with attenuation correction. The PET images were intensity normalized based on injected activity and body weight to derive standard uptake values (SUVs).

The reconstructed PET transaxial image sizes are 168 × 168 matrices (voxel size 4.07 mm × 4.07 mm × 3 mm) and 512 × 512 matrices (voxel size 0.97 mm × 0.97 mm × 3 mm) for CT images. The transaxial PET/CT image extent ranged between 274 and 474 planes per volume, depending on the subject’s height. Initially, a low-dose CT scan was conducted using settings of 110 kV_P_, covering the region from the skull to the mid-thigh. Subsequently, PET imaging was carried out over the same anatomical area, with scan times lasting 3 or 4 min per bed position, dependent on the patient’s weight.

The pre-training dataset included 652 [^68^Ga]Ga-PSMA-11 PET/CT images, compromising both lesion-positive and lesion-negative cases. Additionally, 100 independent labeled images were used for fine-tuning and model evaluation. This dataset included [^68^Ga]Ga-PSMA-11 PET/CT images collected from 100 lesion-positive patients. Comprehensive patient characteristics, including PET/CT indications and patient demographics, are provided in Supplementary Information S1. All 100 patients had clinical reports indicating the presence of tumoral lesions. The included patients in the study had a mean age of 68.8 ± 8.29 years (range of 41–93 years) at the imaging time. All images were formatted to NifTI format.

### Image segmentation and labels

In the 100 images examined during fine-tuning and model evaluation, lesions and OARs were carefully delineated. This process was conducted by a nuclear medicine physician with over five years of proficiency in hybrid imaging and a background in machine learning research using a visually determined threshold for each patient. Another expert cross-checked these delineations to ensure accuracy. The segmentation of OARs and lesions involved converting them into 3D masks with dimensions matching those of the PET and CT images. Bilateral organs combined into a single class (e.g., the left and right kidneys were labeled as “kidneys”), resulting in 10 OARs, lesions, and backgrounds for automated segmentation. Six bilateral segmented structures contained the lacrimal glands, parotid glands, submandibular glands, tubarial glands, sublingual glands, and kidneys. Four unilateral segmented structures included the spleen, liver, bowel (small and large), and bladder.

We collectively categorized primary tumors (if not surgically removed), lymph node metastases, bone metastases, and visceral metastases under the label “lesions”. A contour was manually delineated around OARs and lesions using the “Segment Editor” tool of the free, open-source 3D Slicer 5.2.2 software. The “PETTumorSegmentation” module within the “Segment Editor” was employed for tumor lesion segmentation with manual interventions and corrections. This module uses a semi-automated approach that transforms the segmentation problem into a graph-based optimization problem. This module constructs a graph structure around a user-provided lesion center point, and a suitable cost function is derived from local image statistics [1]. To ensure precision and accuracy, another physician reviewed cases of ambiguity.

Among the OARs, the kidneys, liver, and spleen were delineated using CT-only slices. In this regard, kidneys were segmented using a visually selected threshold [35]. Transversal slices were manually refined to include the cortex and medulla, while excluding the renal pelvis, vessels, cysts, and adjacent structures. For liver and spleen delineation, ROIs were manually drawn once every two to three slices, and the slices were interpolated, and our physician refined the results. PET-only slices were used to segment the lacrimal and tubarial glands because of their small sizes.

**Fig. 1:**
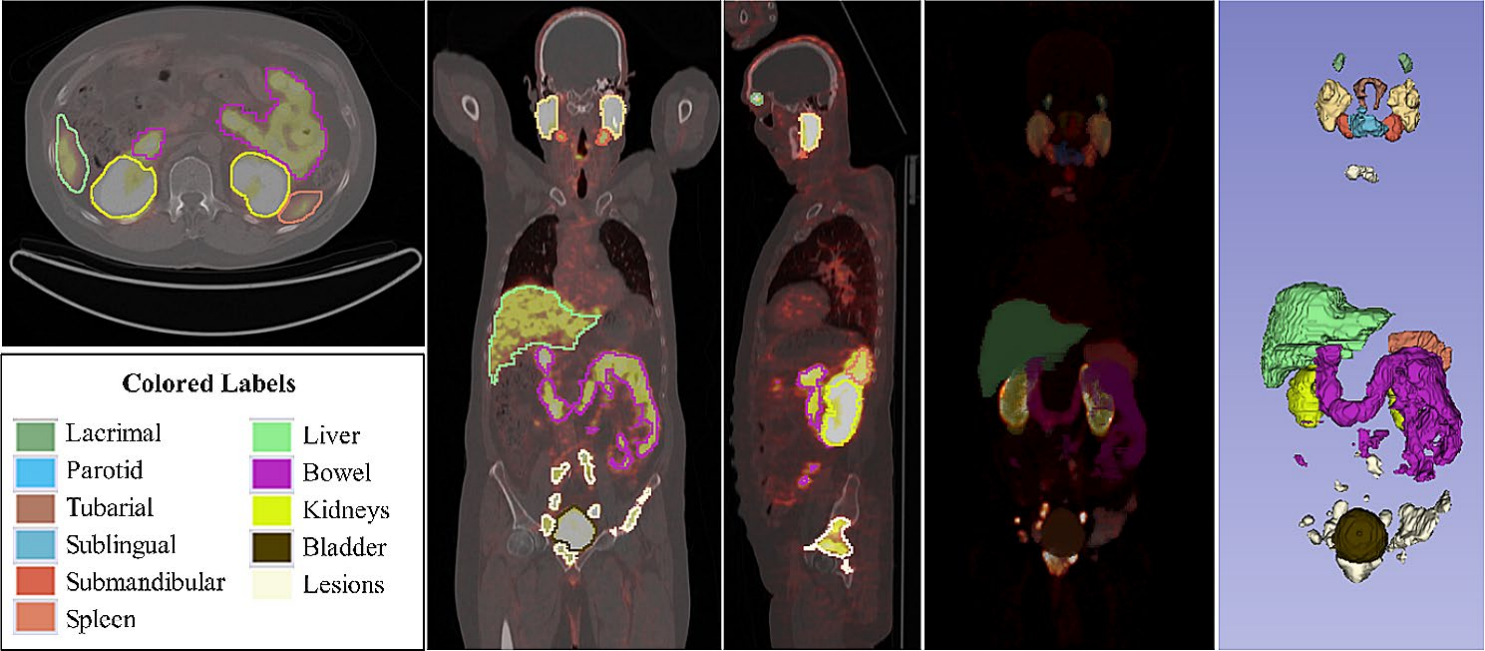
[^68^Ga]Ga-PSMA-11 PET/CT image of a mCRPC patient with segmented targets in transaxial, coronal, sagittal, MIP, and 3D views from left to right using 3DSlicer 5.2.2 software

Due to the highly variable uptake intensity of bowel structures, the absence of a reliable semi-automated method necessitated visual threshold segmentation. To ensure accurate results, our physician carefully analyzed and segmented PET and CT slices, and the second physician was included in cases of disagreement. The same approach was also applied for bladder and bulk prostate tumors. Figure 1 displays a whole-body [^68^Ga]Ga-PSMA-11 PET/CT scan. It shows segmented OARs and lesions in various views - transaxial, coronal, sagittal, maximum intensity projection (MIP), and 3D - all achieved using the 3D Slicer software.

### Data preprocessing

The data was preprocessed and adapted to meet the input requirements of the model as part of the standardization process. CT slices were down-sampled using B-Spline interpolation to align with the coordinates of their corresponding PET images. This process resulted in both CT and PET images having the exact dimensions (168 × 168) and voxel size (4.07 mm × 4.07 mm × 3 mm). The anatomical alignment between PET and CT images was maintained. Three slices from the beginning and end of the PET image series were removed to reduce noise caused by low count accumulation in the first and last slices due to decreased sensitivity in the PET scanner’s outer rings.

The PET intensity values were expressed using an SUV, while the Hounsfield unit or HU was employed for CT intensity values. Typically, PET voxel values fell within the mean range of 0 to 50. Considering the urinary clearance of the radiotracer, the highest SUV_max_ was noted in the kidneys and bladder. For CT images, a window level of 400 and a window width of 600 were considered, and the HU values were clipped between − 200 and 1000 HU to enhance the contrast of soft tissues and lesions (based on consultation with the nuclear medicine physician). Subsequently, CT and PET values were normalized between 0 and 1 using Eq. 1.

1 $$ {X}_{norm}=\frac{(X-{X}_{min})}{({X}_{max}-{X}_{min})}$$

### Network architecture

**Fig. 2:**
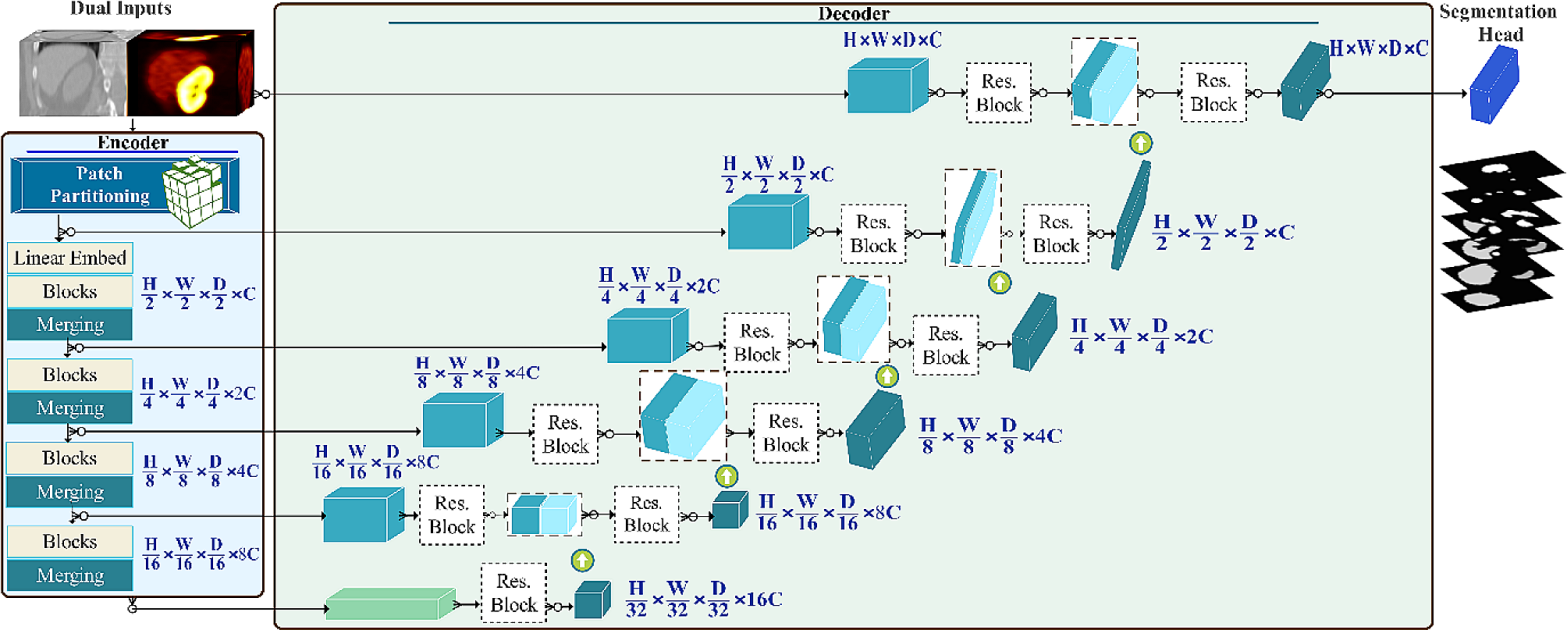
Architecture of the Swin UNTER used in this study. The PET/CT input images undergo processing in the encoder, consisting of four stages, with each stage connected to the decoder through skip connections. Within the encoder, the dimensions of the images progressively decrease at each stage until reaching the bottleneck. Subsequently, in the decoder, the dimensions of the features increase as they ascend through deconvolution layers. The network guidelines are outlined in Supplementary Fig. S1

This study used the Swin UNETR model as illustrated in Fig. 2, which uses a Swin Transformer as its encoder, as described in reference [36]. This hierarchical vision transformer employs shifted windows to capture local and global information. It incorporates a CNN-based decoder with skip connections at different various resolutions. To determine the optimal configuration, we conducted extensive experiments varying sub-volume size, patch size, and window size. Remarkably, the most effective configuration we found involved relatively minor adjustments compared to the original configuration [36]. The following provides a concise description of the encoder and decoder components.

### Encoder

The encoder processes a sub-volume of the image, employing a patch portioning layer that divides the input into non-overlapping 3D tokens of size H×W×D. These patches are then transformed into a sequence with dimension C (here, C = 48). For efficient modeling of token interactions, the partitioning stage divides tokens into non-overlapping windows, and self-attention is then computed among the tokens within each window.

At a given layer $$ l$$, in the transformer encoder, windows of size M × M × M was used to divide a 3D token into regions of (H/M, D/M, W/M). Then, at the following layer $$ l + 1$$, these regions are shifted by (M/2, M/2, M/2) voxels. The outputs of layers $$ l$$ and $$ l +1$$ undergo processing through the Swin Transformer block (see Fig. 2 Swin Transformer Block), which has two transformer blocks in sequence. The first block uses window-based multi-head self-attention (W-MSA) to compute self-attention within each window. In contrast, the second block uses a shifted window-based multi-head self-attention (SW-MSA) to compute the self-attention across the shifted windows. A 3D cyclic-shifting method was employed to enhance the efficiency of batch computation for shifted windowing [36].

The encoder operates on patches of size 2 × 2 × 2, with a feature dimension of 2 × 2 × 2 × 2 = 16, considering the input has two channels (PET/CT), which transforms them into a 48-dimensional embedding space using a linear layer. The encoder architecture consists of four stages, each containing two transformer blocks. Between each stage, a patch merger layer decreases the resolution by half. Stage one includes a linear embedding layer and transformer blocks. In addition, a patch merging layer combines patches with a resolution of 2 × 2 × 2, concatenating them to yield a feature embedding with a dimension of 4 C. Subsequently, a linear layer is employed to downsample the resolution by reducing the dimension to 2 C. This process persists in stages two, three, and four, resulting in feature representations at different levels. These hierarchical features are helpful for downstream applications, such as segmentation.

### Decoder

The decoder part of the model is connected to the encoder through skip connections, forming a U-shape architecture for our primary task, image segmentation. Output sequence representations (see Fig. 2 Hidden Features) from five stages are extracted, reshaped, and fed into a residual block, which consists of two post-normalized 3 × 3 × 3 convolutional layers with instance normalization (See Fig. 2 Res. Block). Then, features from each stage are up-sampled through a deconvolution layer and concatenated with processed features from the previous layer. The concatenated features are fed into a residual block with the abovementioned detail. Subsequently, the processed features from the input volume and the encoder output are combined and fed into a residual block. Finally, the segmentation probabilities are computed using a 1 × 1 × 1 convolutional layer with a softmax activation function.

### Pre-training

We adopted a self-supervised pre-training framework described in reference [33]. This approach involved three proxy tasks: image rotation prediction, image volume inpainting, and contrastive coding. These tasks were used to learn meaningful representations of human body organs within images. In the pre-training phase, only the encoder part of the model is pre-trained, and after pre-training, the encoder is combined with the decoder part. A projection head is attached to the encoder during the pre-training phase for each task, three heads at all. Subsequently, these projection heads are removed during the fine-tuning phase, and the entire model is fine-tuned on label data.

During the training phase, sub-volumes are randomly cropped from the input image. All data points in a mini-batch undergo stochastic data augmentations, which include two transformations: random rotations and cutouts. This process generates two distinct views of the same sub-volume, enhancing the model’s learning capacity. The pre-training loss function calculation is described in the supplementary information S2.

### Experiments

The experiments were conducted in five phases. In the first two experiments, we utilized nnU-Net, a widely recognized network for medical image segmentation tasks, to conduct PET-only nnU-Net and PET/CT nnU-Net experiments. This approach allowed us to benchmark and compare our proposed network against the established results obtained using nnU-Net. For the subsequent three experiments, the proposed model was initially trained using only the PET imaging. Subsequently, both PET and CT images were incorporated for training. These phases involved training the models with random weight initialization and without self-supervised pre-training. In the last experiment, we used pre-trained weights obtained through self-supervised pre-training and fine-tuned the model on our labeled data.

During the pre-training phase, among 752 whole datasets, the encoder was trained on 652 unlabeled PET/CT images (318 from center A and 334 from center B). In the fine-tuning phase, the model was fine-tuned on 100 remainder labeled images (90 from center A and 10 from center B) using dice + cross-entropy (DiceCE) loss function (Eq. 2), where P and G represent the predicted segmentation and ground-truth segmentation, respectively. To assess the model’s performance, we set aside 20 images, of which 10 belonged to center A and the remaining were from center B, as an independent test set. The remaining 80 images were used for training and a 5-fold cross-validation approach was employed. In each fold, the data was divided into 64 images for training and 16 images for testing. Cross-validation was used to select and evaluate the best model on the independent test dataset.

2 $$\eqalign{ DiceCE & = Dice\,Loss + CE \cr & = \left( {1 - {{2\sum\nolimits_{i = 1}^N {{P_i}{G_i}} } \over {\sum\nolimits_{i = 1}^N {{P_i}} + \sum\nolimits_{i = 1}^N {{G_i}} }}} \right) \cr + & \left( { - {1 \over n}\sum\nolimits_{i = 1}^N {\sum\nolimits_{k = 1}^K {{P_{i,k}}{G_{i,k}}} } } \right) \cr} $$

In our pre-training phase, 30% of 3D volumes were masked out in the volume inpainting task. For 3D contrastive coding, an embedding size of 512 was employed, and the rotation prediction task involved four classes corresponding to angles of 0, 90, 180, and 270 degrees. The AdamW optimizer was utilized with a warm-up cosine scheduler for the first 500 iterations and training was conducted for 250k iterations [37]. A batch size of 4 with a patch size of 96 × 96 × 96 was used, alongside an initial learning rate of 1e-6 and a learning decay rate of 1e-5.

The learning rate was adjusted to 1e-4 during the fine-tuning, and the model underwent extensive training for 1000 epochs. We employed PyTorch 1.13 and MONAI 0.9 libraries to implement the models. All models were trained on NVIDIA RTX 3090 GPU with 24 GB GPU memory.

### Evaluation metrics

To assess our model’s performance, dice similarity coefficient (DSC), recall, and precision metrics were used. Further details, including the formulas and definitions of each metric, can be found in the supplementary information S3.

## Results

Table 1 summarizes the averaged DSC values per-patient on the test set for each target in five distinct experiments (the best values in each row and mean values in the last row are emphasized in bold). These experiments utilized the nnU-Net model with two configurations, PET-only and PET/CT for comparative analysis, as well as three configurations for our proposed method: PET-only and PET/CT trained without self-supervised pre-training, and PET/CT with self-supervised pre-training and fine-tuning. The same experiments for all targets are summarized in Tables 2 and 3 for precision and recall metrics, respectively (the best values in each row and mean values in the last row are emphasized in bold). The incorporation of dual inputs in the network significantly improved DSC for all targets compared to using only PET volume, regardless of whether using the nnU-Net or our proposed method. In this regard, the most substantial improvements were observed in the lacrimal glands, spleen, liver, kidneys, bladder, and lesions. In addition, our proposed method exhibited superior results across all metrics when compared to nnU-Net, in the context of both PET-only and PET/CT results. Consequently, the dual input configuration was kept in the last experiment. Moreover, pre-training and fine-tuning with PET/CT inputs yielded improved results compared to training the model without self-supervised pre-training. The recorded DSC values as our primary metric ranged from 0.68 for lesions to 0.95 for the liver, highlighting the model’s efficacy. In summary, the mean DSC values for the five experiments were 0.75, 0.79, 0.77, 0.81, and 0.84, respectively, demonstrating a consistent and noteworthy improvement across all targets.

**Table 1:** The averaged DSC metric results per-patient in five distinct experiments across all targets on the test set

	Dice similarity coefficient				
Approach Target	**nnU-Net - PET**	**nnU-Net -PET/CT**	**Proposed method - PET**	**Proposed method - PET/CT (without SSL pre-raining)**	**Proposed method - PET/CT** **(SSL pre-training + fine-tuning)**
Lacrimal glands	0.75	0.78	0.74	0.80	0.83
Parotid glands	0.86	0.88	0.87	0.88	**0.91**
Tubarial glands	0.68	0.69	0.70	0.70	0.75
Sublingual glands	0.52	0.68	0.70	0.73	0.77
Submandibular glands	0.81	0.85	0.84	0.85	**0.88**
Spleen	0.79	0.85	0.82	0.88	**0.90**
Liver	0.88	0.91	0.88	0.93	**0.95**
Bowel	0.71	0.75	0.73	0.76	0.81
Kidneys	0.80	0.85	0.80	0.87	**0.89**
Bladder	0.81	0.83	0.78	0.83	**0.86**
Lesions	0.55	0.61	0.57	0.65	0.68
Mean	**0.75**	**0.79**	**0.77**	**0.81**	**0.84**

**Table 2:** The averaged precision metric results per-patient in five distinct experiments across all targets on the test set

	Precision				
Approach Target	**nnU-Net – PET**	**nnU-Net -PET/CT**	**Proposed method -** **PET**	**Proposed method - PET/CT (without SSL pre-training)**	**Proposed method - PET/CT** **(SSL pre-training + fine-tuning)**
Lacrimal glands	0.63	0.65	0.65	0.73	0.76
Parotid glands	0.80	0.84	0.82	0.84	**0.89**
Tubarial glands	0.63	0.66	0.64	0.64	0.74
Sublingual glands	0.55	0.61	0.66	0.64	0.70
Submandibular glands	0.80	0.80	0.80	0.79	0.84
Spleen	0.79	0.83	0.81	0.86	**0.86**
Liver	0.86	0.88	0.88	0.90	**0.92**
Bowel	0.69	0.73	0.68	0.72	0.78
Kidneys	0.74	0.80	0.76	0.83	**0.84**
Bladder	0.72	0.75	0.73	0.77	0.81
Lesions	0.52	0.58	0.54	0.60	0.65
Mean	**0.70**	**0.74**	**0.72**	**0.75**	**0.80**

**Table 3:** The averaged recall metric results per-patient in five distinct experiments across all targets on the test set

	Recall				
Approach Target	**nnU-Net – PET**	**nnU-Net -PET/CT**	**Proposed method -** **PET**	**Proposed method - PET/CT (without SSL pre-training)**	**Proposed method - PET/CT** **(SSL pre-training + fine-tuning)**
Lacrimal glands	0.89	0.92	0.92	0.91	**0.93**
Parotid glands	0.90	0.94	0.95	0.94	**0.95**
Tubarial glands	0.81	0.85	0.84	0.84	0.81
Sublingual glands	0.78	0.81	0.83	0.90	0.89
Submandibular glands	0.86	0.90	0.92	0.94	**0.94**
Spleen	0.82	0.89	0.83	0.91	**0.96**
Liver	0.89	0.95	0.89	0.96	**0.99**
Bowel	0.81	0.86	0.84	0.86	0.86
Kidneys	0.85	0.90	0.86	0.92	**0.94**
Bladder	0.82	0.91	0.86	0.93	**0.94**
Lesions	0.63	0.68	0.70	0.78	0.78
Mean	**0.82**	**0.88**	**0.86**	**0.90**	**0.89**

In Tables 2 and 3, precision and recall exhibited an increase from the first to the second experiment, indicating the superior performance of PET/CT nnU-Net over PET-only nnU-Net. Moreover, the comparison between the first and third-column experiments in both tables highlights the enhanced performance of our proposed network. This distinction becomes more apparent when comparing the results of the second experiment with those of the fourth across all targets.

Comparing the last two columns in Table 2 for the precision metric indicates that results obtained through self-supervised pre-training outperform those obtained from training PET/CT segmentation from scratch with random weight initialization. However, Table 3 demonstrates that the dual input PET/CT without a self-supervised pre-training experiment achieved the highest recall rates. A significant agreement was found between manual and automated segmentation based on the quality metrics. This agreement was particularly evident in large organs with high tracer uptake, such as the liver, kidneys, parotid glands, and spleen. The most noticeable differences between manual and network segmentation were observed in the tubarial, sublingual, and lesion regions.

**Fig. 3:**
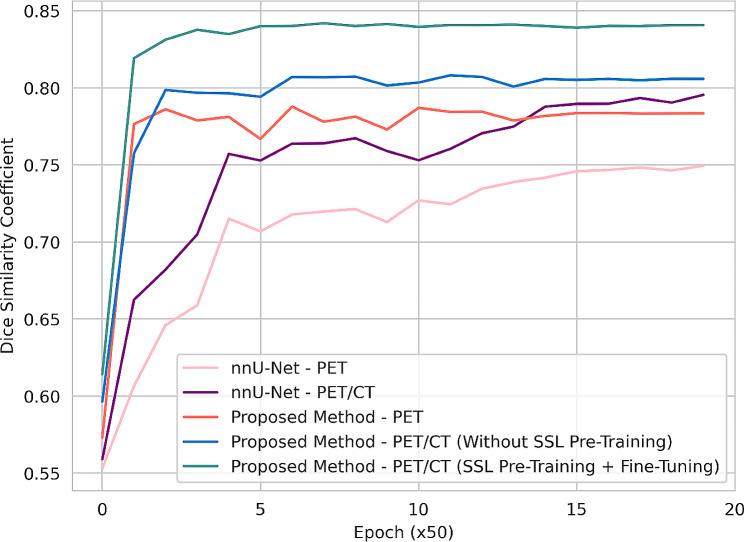
Comparing five experiments using the mean DSCs for each 50 epoch in the validation mode

According to Fig. 3, the DSCs were calculated for every 50 epochs in the validation mode. The figure shows the PET-only nnU-Net experiment in pink, PET/CT nnU-Net in purple, and the three experiments involving the proposed network: PET-only in orange, PET/CT trained without self-supervised pre-training in blue, and PET/CT with self-supervised pre-training and fine-tuning in green. Figure 3 illustrates that the last experiment starts with a higher DSC in the initial epochs, surpassing 80% around epoch 100. Additionally, the stability of the experiment is also higher than that of the other experiments, and it lacks the oscillations observed in the other experiments.

**Fig. 4:**
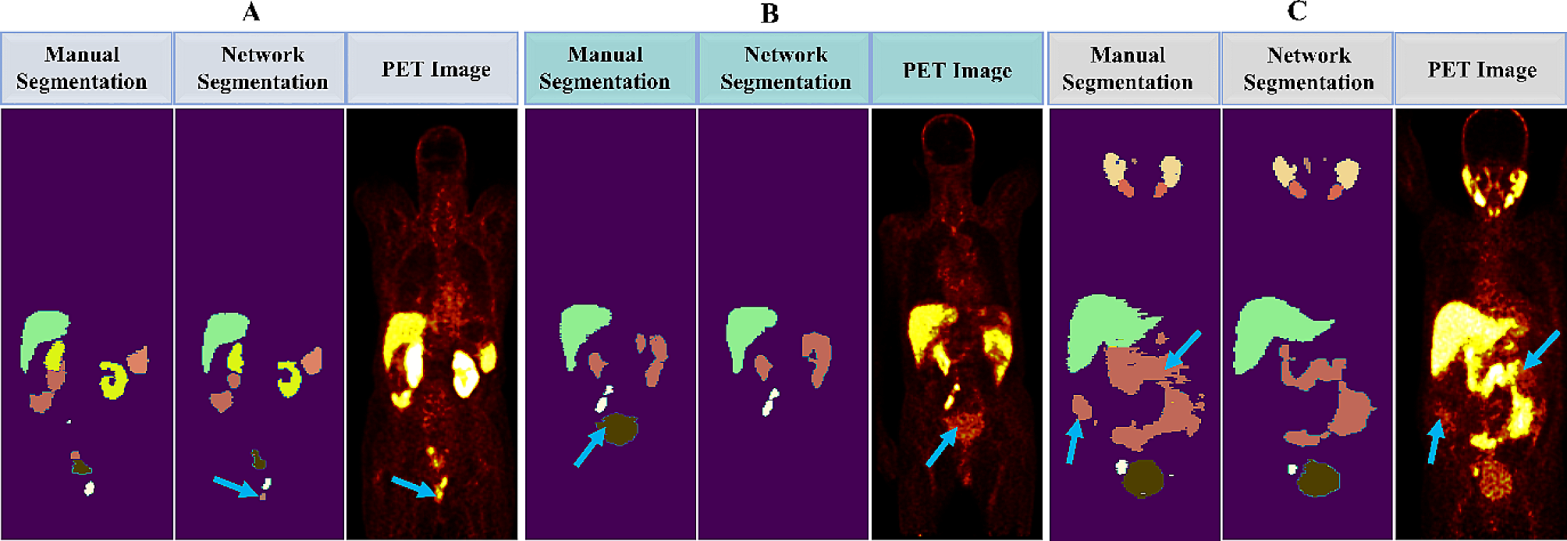
Coronal views illustrate mismatches in manual segmentation vs. automated OAR segmentation. **(A)** High-activity urine in the urethra was mislabeled as a tubarial gland. **(B)** Network failure in bladder segmentation due to lower activity caused by renal failure. **(C)** Network failed to segment bowel in low activity regions

A less frequent error was observed in the automatic segmentation of urine in the ureter region with high SUV values, where the network was occasionally misclassified. The network incorrectly classified a cluster of voxels associated with urine near the bladder as a tubarial gland, as shown in Fig. 4A by an arrow. There was another discrepancy between automatic and manual segmentation for renal failure patients. The network failed to detect the bladder because of minimal urinary activity. For instance, as shown in Fig. 4B, the network did not segment the bladder where the renal failure results in a SUV < 3. One noteworthy observation was bladder segmentation, even with a SUV < 3 in patients with healthy renal function. This is evidenced by the case illustrated in Fig. 4C, where the bladder was accurately segmented. Despite this, the network failed to include the lower-activity regions in the bowel of the same patient in Fig. 4C, as shown by the arrows. The challenge is because of the complex nature of bowel structures and varying uptake intensity.

**Fig. 5:**
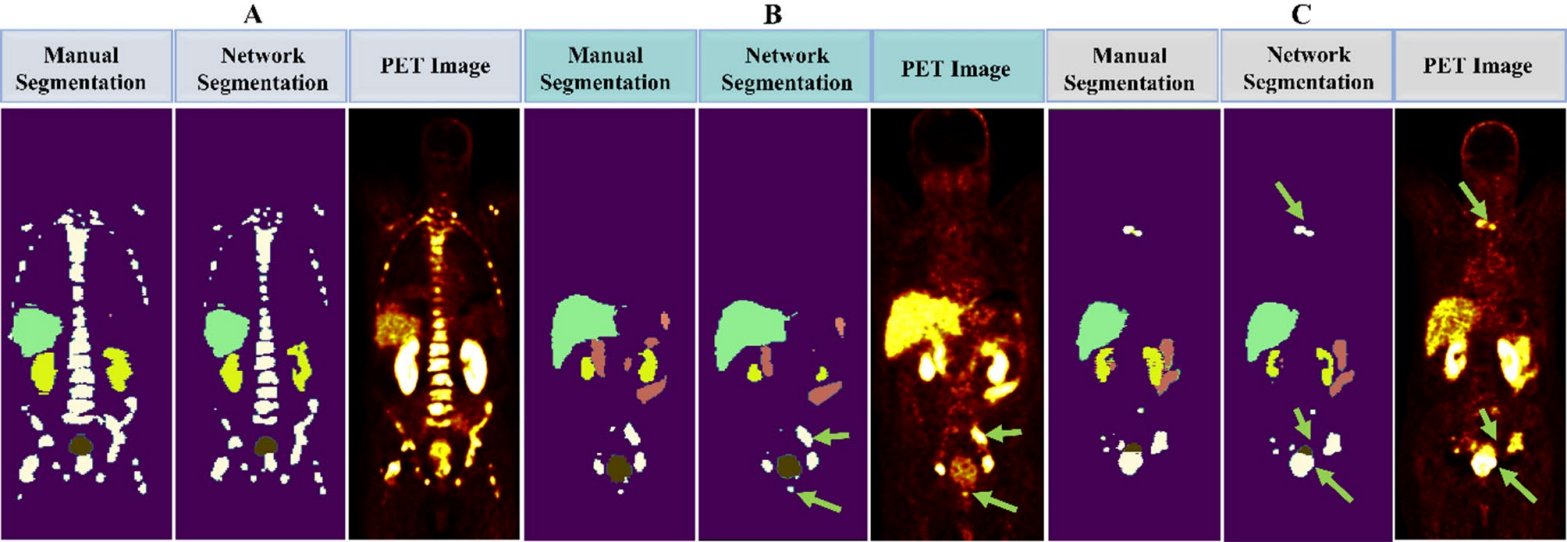
Coronal views illustrate the network’s proficiency in segmenting various lesions. **(A)** widespread axial and appendicular bone metastases. **(B)** Regional lymph node metastases. **(C)** Regional tumoral extension (tumor invasion to the bladder wall) and simultaneous regional and distant metastases

In Fig. 5A, the network successfully delineates widespread axial and appendicular bone metastases. Moreover, Fig. 5B presents an accurate automated segmentation of regional lymph node metastases, as indicated by arrows. In Fig. 5C, the network successfully identifies the invasion of the tumor into the bladder wall, along with regional and distant metastases. As seen in the same figure, alongside lymph node lesions and a prostate tumor, the bladder is well segmented by the network. These examples underscore the network’s effectiveness in handling complex lesion patterns.

**Fig. 6:**
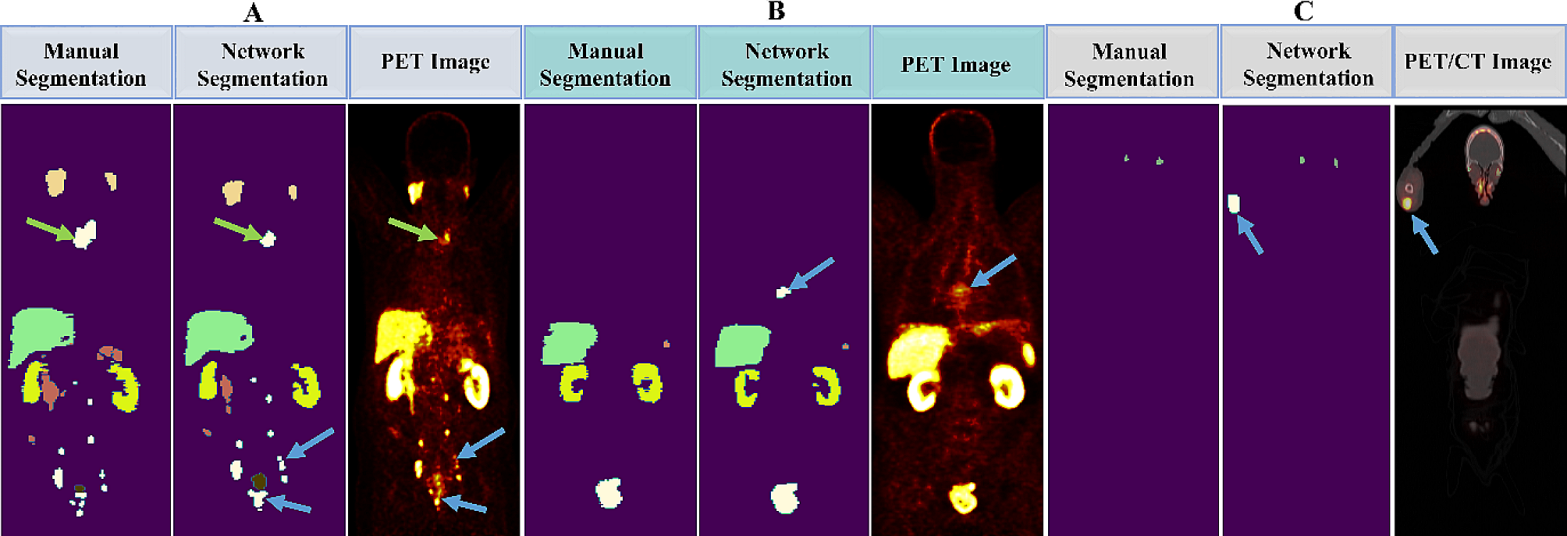
Coronal views illustrate mismatches between manual segmentation and automated lesion segmentation by the network. **(A)** The network considered additional (blue arrows) or fewer (green arrow) lesion voxels. **(B)** The network misclassified liver inflammation as a lesion. **(C)** The network mistakenly categorized the injection site as a lesion

The main difference between manual delineation and automatic lesion segmentation, as seen in Fig. 6A, is the network’s inclusion of high-activity voxels that were unintentionally missed during manual segmentation (blue arrow). On the other hand, the automatic process sometimes overlooked less-active lesion voxels (with SUV < 3) that were manually identified (green arrow). Moreover, the network considers lung inflammation a lesion, even though it has not been manually segmented, as shown in Fig. 6B. Furthermore, there were cases where the injection site in hand led to high-activity voxels, and the network incorrectly labeled them as lesions, as illustrated in Fig. 6C.

## Discussion

Manually segmentation of metastatic lesions in whole-body PSMA PET/CT images of mCRPC patients with multiple or disseminated metastases is impractical due to the extensive time and expertise required for each case. The variability in the lesions’ shapes, sizes, and radiotracer uptake levels further complicates this task. However, recent advancements in deep neural networks present a promising opportunity to automate this segmentation process, given adequate data for model training [38]. Additionally, several semi-automated segmentation methods (e.g., aPROMISE, qPSMA, MIM, etc.) have been proposed for quantification, but have not been widely adopted [39–42]. For instance, the qPSMA tool utilizes a liver uptake-based threshold for lesion selection in advanced prostate cancer patients. However, as many lesions have SUV_max_ values below the threshold of mean liver uptake, such methodologies struggle to detect PSMA avid disease. Rigid threshold-based segmentation, such as 50% or 30% of lesion SUV_max_, may inaccurately over-segment lesions, especially those with subtle or low uptake [39, 40]. The aPROMISE tool, which was FDA-cleared and CE-marked in 2021, employed a U-net architecture and provided quantitative analysis and standardized reporting for PSMA PET/CT scans; however, it exhibited a relatively high number of false-positive lesions per-patient. The article did not specify if manual corrections were necessary, and aPROMISE lacks independent validation [40, 41]. MIM software is widely used for 3D PET image analysis in various medical applications, such as oncological diagnostics, therapy, neurology, and radionuclide dosimetry [42–44]. However, its complexity and potential cost could pose challenges, especially for users seeking a more user-friendly interface or for institutions with budget constraints. Therefore, it is critical to address these issues before AI can be considered clinically valuable.

There is a substantial uptake in the lesions in different anatomical regions, frequently close to healthy organs. Tumors and metastasis can occur with unpredictability and heterogeneity, whereas the spatial information of OARs with physiological uptakes remains stable. Given this stability, segmentation of OARs on PET/CT images can be a preliminary step toward automating tumor segmentation. For routine implementation of quantitative PET image analysis, RPT planning, and radiomic analysis, a comprehensive PET/CT model capable of segmenting both OARs and lesions is essential [45]. Despite the significant potential of AI to segment PET and PET/CT images in oncology automatically, supervised techniques face significant challenges because of the need for more consensus on manual delineations and inter- and intra-observer variabilities [24]. Transformer-based models learn more accurate feature representations than CNN-based counterparts during pre-training and perform better on downstream tasks that require fine-tuning. Several approaches, such as semi-supervised, neuro-symbolic AI, federated learning, and self-training frameworks, are actively being explored to deal with limited (annotated) data [24].

We proposed utilizing the Swin UNETR model with transfer learning to automate the segmentation of whole-body [^68^Ga]Ga-PSMA-11 PET/CT images of lesion-positive patients, including mCRPC [36]. To this aim, we undertook extensive experiments, including variations in sub-volume size, patch size, and window size, to determine the best configuration that yielded superior results. In five distinct experiments, the effectiveness of the network was compared for PET-only nnU-Net, PET/CT nnU-Net, PET-only proposed method, PET/CT inputs (without self-supervised pre-training), and PET/CT inputs with both self-supervised pre-training and fine-tuning. For testing the robustness of the DL methods to slight differences in the protocol, PSMA PET/CT data from the two centers were included. Centers A and B provided 408 (318 for self-supervised pre-training and 90 for fine-tuning) and 344 (334 for self-supervised pre-training and 10 for fine-tuning) databases of PSMA PET/CT images, respectively.

Ten OARs and lesions were manually segmented from [^68^Ga]Ga-PSMA-11 PET/CT images of 100 lesion-positive patients, including mCRPC. Additionally, 652 images, comprising both lesion-positive and lesion-negative cases, were left without segmentation for use in self-supervised pre-training of the model. The test dataset consisted of 20 subjects (10 from each center) for supervised training. A 5-fold cross-validation was conducted using the remaining data, where each fold consists of 64 subjects for training and 16 for testing. The data was augmented by introducing random rotations, random flipping, and cropping the foreground to enhance the segmentation quality.

With the recent FDA approval of [^68^Ga]Ga-PSMA-11 and other rapid advances in PSMA imaging, our segmentation model holds promise for improving the treatment of mCRPC patients by making image information more accessible [46, 47]. In this regard, we can calculate precisely the amount of radiation delivered to healthy organs during [^177^Lu]Lu-PSMA RLTs as the therapeutic pair of [^68^Ga]Ga-PSMA-11. According to Tables 1, 2, and 3, in PET-only experiments, the proposed network outperforms the well-known and widely applied nnU-Net in the segmentation tasks. The segmentation accuracy notably increases when trained with PET/CT compared to PET-only, regardless of whether using nnU-Net or our proposed network. The impact of pre-training on the convergence of network training is illustrated in Fig. 3, depicting validation losses across every 50 epochs. According to the plots, our proposed network has superior DSC compared to the widely-used nnU-Net method for medical image segmentation tasks, whether utilizing PET-only or PET/CT images. This indicates the effectiveness of the Swin Transformer when used as the encoder to capture local and global information in image segmentation task. The figure highlights that without pre-training, validation loss tends to be unstable. The incorporation of self-supervised pre-training not only enhances the rate of loss minimization but also conveys stability and monotonicity behavior in the validation loss. Furthermore, self-supervised pre-training and fine-tuning yielded a super-additive improvement in accuracy in terms of DSC and precision. Self-supervised pre-training significantly improved training convergence time and segmentation quality by potentially encoding anatomical and functional features priors in the network, such as organ shapes and spatial relationships. As shown in Table 1, considering the last experiment, the average lowest and highest DSC values per-patient were 0.68 for the lesions and 0.95 for the liver. The mean DSC among OARs without considering lesions averaged about 0.86, and with considering lesions, it was approximately 0.84.

Notably, there were no observed improvements in recall when applying self-supervised pre-training, particularly in small targets, such as lesions and small glands. It is worth emphasizing that DSC is the most commonly used metric for validating medical volume segmentations and has become a golden standard for assessing the segmentation quality from a voxel-based perspective [48]. However, recall and precision are not as commonly considered as the primary metrics in the medical image segmentation evaluation due to their sensitivity to segment size, penalizing errors in small segments more than in large ones [48]. Therefore, evaluating the model solely based on precision or recall is not reasonable. Given the unique requirements of lesion segmentation tasks, alternative metrics, such as DSC may be more suitable to evaluate the efficacy of a novel method. In this regard, we considered DSC as the primary metric for evaluating the impact of pre-training through a direct comparison between automatic and ground truth segmentations.

Regarding comparing the results with other studies, we should note that FDG PET scans have been the focus of most AI segmentation techniques, while other radiotracers have rarely been considered [49–51]. Using a 2.5D U-Net architecture, Zhao et al. [22] segmented prostate lesions, lymph nodes, and bones with localized and secondary prostate tumors from [^68^Ga]Ga-PSMA-11 PET/CT images. They achieved average DSCs of 64.5% for bone lesions and 54.4% for lymph node lesions on dual input PET/CT. Xue et al. [52] developed a U-Net-based framework in whole-body PET/CT images with the [^18^F]DCFPyL radiotracer to automatically segment metastatic prostate cancer lesions. The authors proposed to use weighted batch-wise dice loss and achieved an average median lesion-wise DSC of 0.51 and 0.60 for lesions with SUV_max_>5.0. A review of AI techniques for tumor segmentation in PET and CT images was conducted by Yousefrizi et al. [24], emphasizing the need for clinical integration of these techniques.

In terms of OAR segmentation, Leube et al. [35] applied five different u-net-based approaches to automatically segment kidneys in 53 [^68^Ga]Ga-PSMA-I&T and 55 [^18^F]PSMA-1007 PET/CT examinations. It is not appropriate to compare the results, since the differences in tracers make it impossible to compare their DCS of 0.93 for kidneys with ours of 0.89. A noteworthy observation was found regarding the kidneys, as shown in Fig. 4B. The network failed to segment the bladder of a patient with renal failure. In contrast, the kidneys segmented significantly with a DSC of 0.92.

Using deep neural networks, Toosi et al. [53] automated the segmentation of salivary and lacrimal glands from [^18^F]DCFPyL PET/CT images and achieved a mean DSC of 0.87. Salivary and lacrimal glands are dose-limiting OARs in PSMA-RLT. Radiopharmaceutical uptake in these OARs could estimate radiation dose pre-treatment, help select an optimal dose, and prevent severe side effects. In our work, the tubarial and parotid glands had the lowest and highest DSC, respectively, and the mean DSC among all the glands was 0.83. Using multi-target CNNs, Klyuzhin et al. [54] automatically segmented all OARs on [^18^F]DCFPyL PET/CT images. Their model achieved the lowest and highest mean DSCs for the parotid glands and the bladder at 0.86 and 0.90, respectively. The authors found that a multi-organ network is more effective than a single-organ network if they have similar architectures, so in the present work, only the multi-target network was considered. In contrast to other studies, such as those mentioned above, our study focuses on both lesion and OAR segmentation.

The results indicated that automatic segmentation can be more accurate than manual segmentation by a nuclear medicine physician, especially for bowel and lesion segmentation. Therefore, the network could handle some inconsistencies in training and testing. Given the high anatomical variability and diverse uptake patterns of the bowel, its DSC was relatively low, around 0.8. According to Fig. 4C, the network struggles with segmenting the region of the intestinal tract with low activity.

The network precisely segmented lesions that are not uniformly distributed across patients; some patients may have an abundance of lesions in bone and lymph nodes (see Fig. 5A), while others may suffer only from isolated lesions (see Fig. 5B and C). An interesting finding in our study was strong model performance on lesions close to the bladder, such as the cases depicted in Fig. 5. The network accurately segmented different lesions, such as bone and lymph node metastases, as well as prostate tumors.

Poor performance is likely because of high image noise levels and patient PSMA expression variations. In another notable error in lesion segmentation, the physician included more voxels around a detected lesion (Fig. 6A, green arrow). Sometimes, the physician left some lesion voxels with high uptake around the lesion site (Fig. 6A, blue arrow). As shown in Fig. 6B, one false positive error by the network is detecting the uptake of lung inflammation because of enthesopathy as a lesion. If there is a slight overlapping by the bone around this region, it can increase this error. In our study group, one differentiated liver met was found, which was insufficient for our network training and, therefore, was one source of error in network lesion detection. Another minor source of error in both manual segmentation and network segmentation is focal urinary stasis in the ureter. However, our network performance was acceptable in this region; these parts need more attention during manual segmentation. As shown in Fig. 4A, the network labeled the focal urinary as a tubarial gland. In some other cases, it labeled the same region as lesions. Also, active urine in the urethra or urinary contamination on the skin was another source of errors. The guidelines suggest reducing urinary bladder activity to enhance the accuracy of diagnosing adjacent lesions [46]. Automated lesion detection can be further improved by adopting a revised protocol during data collection.

Conglomerated lymph nodes in the retroperitoneal region with extension to the intraperitoneal cavity mimic radiotracer uptake in the bowel. Considering the rarity of this case, the network was not fully trained to find these regions, ultimately leading to another source of error. The last point in lesion detection is that the focal radiotracer uptake at the injection site is a source of false positive error (see Fig. 6C). To overcome this error, the injection site should be segmented separately in future studies.

The proposed approach might simplify diagnostics and therapeutics studies by automatically segmenting radiopharmaceutical accumulation in OARs and lesions. Dual-input PET/CT images were incorporated to enhance the detection and classification of OARs and lesions. According to the manual interpretation of the fusion images, PSMA PET/CT significantly improved the detection and characterization of tumor lesions and OARs [55].

Our contribution lies in introducing a novel approach that has not previously been applied to PSMA-PET/CT imaging. We believe that our method has the potential to affect clinical practice due to its state-of-the-art architecture. This study may provide insights into future research directions and improvements in detecting whole-body tumor burdens, potentially paving the way for further research and refinement. With a self-supervised pre-training and supervised fine-tuning approach, our method achieves a remarkable 5% improvement in DSC, surpassing that of the nnU-Net with PET/CT input. Moreover, as a result of the collaboration between the two centers in this study, the algorithm has the potential to be applied to other centers, thereby likely accelerating its clinical translation. A key contribution of our study is the utilization of self-supervised learning for PSMA-PET/CT imaging. With pre-training on unlabeled data (652 samples), we demonstrated a 3% improvement in accuracy and reduced training time for significant performance. Moreover, our method is scalable, as incorporating additional unlabeled data for pretraining could further enhance the results. This scalability is particularly feasible in clinical settings where regular PET/CT images are acquired, alleviating the need for clinicians to manually annotate pre-training images. To facilitate further evaluation, we have shared the source code of our network on GitHub. This allows the model to be tested on other datasets by researchers and clinicians, promoting generalization and collaboration.

### Limitations and future perspectives

The proposed network demonstrated moderate performance in delineating lesions of different sizes and uptake patterns; however, further improvement is required before it can be applied in clinical practice. Various factors may contribute to the DSC of 0.68 for lesions, including the limited size of training sets and significant variability in lesion size, shape, proximity to organs, and intensity of uptake. While it is possible to further categorize the tumor burden into different categories such as local, bone, and lymph node, it is essential to note that most lymph node metastases commonly occur in the pelvic region, which is known as an indicator of prognosis [56, 57]. Future research can focus on differentiating lymph node metastases in the pelvic and extrapelvic sites (such as paraaortic, mediastinal, and interaortocaval), as well as investigating the involvement of bone metastases.

In this study, we evaluated the segmentation efficiency of the Swin UNETR considering per-patient measurements; however, per-lesion measurements could provide further insights. Moreover, we found that the pre-trained model used in our segmentation task performed well in lesion-positive subjects; however, it requires generalization. Potential solutions include utilizing multi-modal data that includes metadata (such as disease history) or patient medical reports, implementing effective signal-noise discrimination through noise modeling, or training on a larger labeled dataset with greater diversity in disease staging, including healthy cases. Annotating the data for an extensive database is still challenging due to logistical difficulties, necessitating the substitution of manual labeling. Enhancing annotation efficiency and reliability can be achieved through an iterative process that combines automatic annotation with manual editing.

Although interobserver differences in delineation are known, we used the manual delineation as a reference standard [58]. In addition, human-generated delineations of organs usually include some level of inconsistency. It was observed that automatic segmentations were at times more accurate than physician-generated segments, particularly in the bowel and lesions. The network generated more consistent segmentations on the test set than a physician, as it could address some inconsistencies in the training phase. Phantom studies, or studies with consensus segmentations, could help in objectively measuring segmentation performance. It is important to note that the visible lesion boundary may differ from the pathological abnormality location due to partial volume effects [22]. An alternative solution involves training on a vast dataset annotated by diverse experts in the field [59].

While post-processing enhancement techniques can significantly improve segmentations and serve as crucial tools to address inherent algorithm limitations, we intentionally avoided implementing post-processing methods in this study. The primary objective of this study was to examine Swin UNETR’s performance on its own.

DL models have the capability to distinguish between low-level scanner features and high-level patient features, making them more robust and flexible in accommodating cross-domain differences arising from different scanners or protocols. Through collaboration with domain experts, domain-guided proxy tasks can be proposed to facilitate the learning of better data representation for downstream tasks, such as segmentation of PET/CT images. Moreover, a similar model can be developed for theranostics PSMA SPECT/CT images, focusing on optimizing the model for lower-resolution SPECT/CT images. The automated detection and segmentation of lesions are crucial for enhancing treatment planning and monitoring responses in radionuclide therapy. The present findings provide an extensive baseline and suggest promising directions for developing automated quantification metrics that are clinically acceptable for enhancing prostate cancer patient outcomes.

## Conclusion

In a nutshell, segmentation plays a crucial role in PSMA imaging clinical tasks, radiotherapy planning, radiomics and dosiomics analyses, and routine dosimetry or dose prediction. This study investigated and tested a neural network for the automated segmentation of 10 OARs and lesions in [^68^Ga]Ga-PSMA-11 PET/CT images. We utilized self-supervised pre-training with the Swin UNETR transformer encoder for fine-tuning to address the lack of annotated data. The highest performance was achieved using PET/CT inputs with self-supervised pre-training and fine-tuning in multi-target segmentation. Lesions and the liver have the lowest and highest DSCs of 0.68 and 0.95, respectively. A substantial amount of training data improves DL methods relevant to whole-body examinations. AI-based segmentation methods for oncological PET images promise to provide personalized cancer treatment.

### Electronic supplementary material

Below is the link to the electronic supplementary material.

**Supplementary Material 1: S1.** Patient characteristics. **S2.** Pre-training loss function calculation. **S3.** Evaluation metrics

**Supplementary Material 2:** Network architecture guideline of Fig. 2

## Data Availability

The datasets analyzed during the current study are not publicly available for information governance reasons. Access requests will be reviewed individually for application to the corresponding author. The code to reproduce the experiments is available at the following URL: https://github.com/ElmiraYazdani/Lesions-OARs-Segmentation-PSMA-PETCT-SSL-SwinUNETR.

## Abbreviations

AI: Artificial intelligence
CNN: Convolutional neural networks
CT: Computerized tomography
DiceCE: Dice + cross-entropy
DL: Deep learning
DSC: Dice similarity coefficient
FN: False negative
FP: False positive
mCRPC: metastatic castration-resistant prostate cancer
MIRD: Medical internal radiation dose committee
OAR: Organ at risk
PET: Positron emission tomography
PC: Prostate cancer
PSMA: Prostate-specific membrane antigen
RLT: Radioligand therapy
ROI: Region of interest
RPT: Radiopharmaceutical therapy
SSL: Self-supervised learning
SUV: Standard uptake values
SW-MSA: Shifted window-based multi-head self-attention
Swin UNETR: Shifted windows UNEt transformers
TN: True negative
TP: True positive
ViT: Vison transformer
VOI: Volume of interest
W-MSA: Window-based multi-head self-attention
