# Supplementary material for: Automated segmentation of lesions and organs at risk on [68Ga]Ga-PSMA-11 PET/CT images using self-supervised learning with Swin UNETR
Source: Cancer Imaging. 2024 Feb 29;24:30. doi: 10.1186/s40644-024-00675-x (PMC10903052; doi:10.1186/s40644-024-00675-x)
Supplement: Supplementary file 1 — Supplementary Material 1: S1. Patient characteristics. S2. Pre-training loss function calculation. S3. Evaluation metrics [file 40644_2024_675_MOESM1_ESM.docx]

**Supplementary Information**

**S1. Patient characteristics**

**Table S1.** Patient Characteristics.

| Variable | | Dataset (752 [^68^Ga]Ga-PSMA-11 PET/CT imaging) | | | | |
| --- | --- | --- | --- | --- | --- | --- |
|  |  | **Self-supervised pre-training set** | | **Training set** | **Testing set** | |
|  |  | **Center A** | **Center B^1^** | **Center A** | **Center A** | **Center B^1^** |
| No. of patients (n) | | 318 | 334 | 80 | 10 | 10 |
| PSA (median, range, ng/mL) | | 8.34,  (0.06-2037) | - | 20.34,  (0.42-2156) | 5.14,  (0.35-559.8) | - |
| Age (mean ± SD, years) | | 68.62 ± 7.12 | 68.93 ± 7.83 | 69.03 ± 8.48 | 69.81 ± 9.13 | 67.61 ± 8.89 |
| PET/CT results | Positive | 191 | 198 | 80 | 10 | 10 |
|  | Negative | 127 | 136 | 0 | 0 | 0 |
| Ga-PSMA PET/CT indications | Staging | 86 (27%) | 79 (24%) | 16 (20%) | 2 (20%) | 2 (20%) |
|  | Evaluation of recurrence/metastasis | 219 (69%) | 241 (72%) | 61 (76%) | 8 (80%) | 7 (70%) |
|  | Other^2^ | 13 (4%) | 14 (4%) | 3(4%) | 0 (0%) | 1 (10%) |

^1^The clinical history of "Center B" was unavailable.

^2^Other indications, including evaluation of response to treatment, restaging, surveillance, etc.

Considering that only mCRPC patients are treated in the nuclear medicine department, we assessed this specific patient group. Hormone-sensitive prostate cancer patients or those with biochemical recurrence were not included in our evaluations. Our primary focus was to document indications for PET/CT scans, encompassing all available scans and patient information, without specifically considering the disease stage.

**S2. Pre-training loss function calculation**

**Masked volume inpainting**

Inspired by [1], inpainting applied to 3D volumes, the cutout augmentation randomly masks out regions of interest within the sub-volume $\chi\epsilon\mathbb{R}^{H\times W\times D\times C}$, with a volume ratio denoted as s. A transpose convolution layer was employed as the reconstruction head attached to the encoder for the reconstruction. The output of this reconstruction process is represented as: $\hat{\chi}^{\mathcal{M}}$. The reconstruction objectives are measured using an L_1_ loss between $\chi$ and $\hat{\chi}^{\mathcal{M}}$ according to Eq. S1.

| $\mathcal{L}_{inpaint}=\left\Vert\chi-\hat{\chi}^{\mathcal{M}} \right\Vert_{1}$. | (S1) |
| --- | --- |

**Image rotating**

The input sub-volume is rotated by a class of 0, 90, 180, and 270 degrees along the z-axis, and the rotation prediction task predicts the class, achieved through an MLP classification head that outputs the softmax probabilities ($\hat{y}_{r}$) corresponding to these rotation classes. With the known ground truth $y_{r}$, cross-entropy loss was used for rotation prediction as defined in Eq. S2.

| $\mathcal{L}_{rot}=-\sum_{r=1}^{R} y_{r}\log\left( \hat{y}_{r} \right)$. | (S2) |
| --- | --- |

**Contrastive coding**

Self-supervised contrastive coding shows considerable promise in visual representation learning, mainly when applied to subsequent tasks [2]. This approach achieves contrastive coding through a linear layer attached to the Swin UNETR encoder, mapping each augmented sub-volume into a latent representation $v$. It learns better representations by maximizing the mutual information between augmented samples from the same sub-volume (positive pairs) while minimizing it between views from different sub-volumes (negative pairs). The 3D rotation and cutout techniques were employed to produce augmented samples from the same sub-volumes. Cosine similarity is employed to measure the distance between encoded representations, as detailed in [2]. For 3D volumes, the contrastive loss is defined as Eq. S3 between a pair of $v_{i}$ and $v_{j}$:

| $\mathcal{L}_{contrast}=-\log\frac{exp(sim(v_{i},v_{j})/t)}{\sum_{k}^{2N} 1_{k\neq i}exp(sim(v_{i},v_{k})/t)}$ | (S3) |
| --- | --- |

Here, t represents the normalized temperature scale measurement. The indicator function, denoted as 1, evaluates to 1 only when $k\neq i$. Meanwhile, $sim$ signifies the dot product of the normalized embedding vectors. The loss function of contrastive learning enhances the intra-class compactness and the inter-class separability.

**Final loss function**

The Swin UNETR’s encoder is trained by minimizing Eq. S4, which is the sum of the aforementioned multiple pre-training objectives as the loss function.

| $\mathcal{L}_{tot}=\lambda_{1}\mathcal{L}_{inpaint}+\lambda_{2}\mathcal{L}_{contrast}+\lambda_{3}\mathcal{L}_{rot}$. | (S4) |
| --- | --- |

**S3. Evaluation metrics**

The following metrics were used to evaluate the model per patient by averaging across all patients, resulting in a detection rate.

In Eq. S5, N represents the total test images. The overlap between predicted segmentation and ground truth masks was determined using the DSC employed in the loss function. The precision metric (Eq. S6) calculates the ratio of true positive (TP) results to all positive ones, which includes both TP and false positive (FP) results. Recall (Eq. S7), also known as sensitivity, measures the ratio of TP outcomes to the sum of TP and false negative (FN) results. Using the confusion matrix, as shown in Eq. S5, the DSC can be expressed alternately based on precision and recall.

| $DSC \left( P,G \right)=\left( \frac{2\sum_{i=1}^{N} P_{i}G_{i}}{\sum_{i=1}^{N} P_{i}+\sum_{i=1}^{N} G_{i}} \right)or DSC=\frac{2TP}{2TP+FP+FN}$ | (S5)  (S6)  (S7) |
| --- | --- |
| $Precesion=\frac{TP}{TP+FP}$ |  |
| $Recall=\frac{TP}{TP+FN}$ |  |

**References**

1. Pathak D, Krahenbuhl P, Donahue J, Darrell T, Efros AA, editors. Context encoders: Feature learning by inpainting. Proceedings of the IEEE conference on computer vision and pattern recognition; 2016.

2. Chen T, Kornblith S, Norouzi M, Hinton G, editors. A simple framework for contrastive learning of visual representations. International conference on machine learning; 2020: PMLR.
