# Supplementary material for: Automated segmentation of lesions and organs at risk on [68Ga]Ga-PSMA-11 PET/CT images using self-supervised learning with Swin UNETR
Source: Cancer Imaging. 2024 Feb 29;24:30. doi: 10.1186/s40644-024-00675-x (PMC10903052; doi:10.1186/s40644-024-00675-x)
Supplement: Supplementary file 2 — Supplementary Material 2: Network architecture guideline of Fig. 2 [file 40644_2024_675_MOESM2_ESM.docx]

**Supplementary Figure**

**Network architecture guideline of Fig. 2.**

**
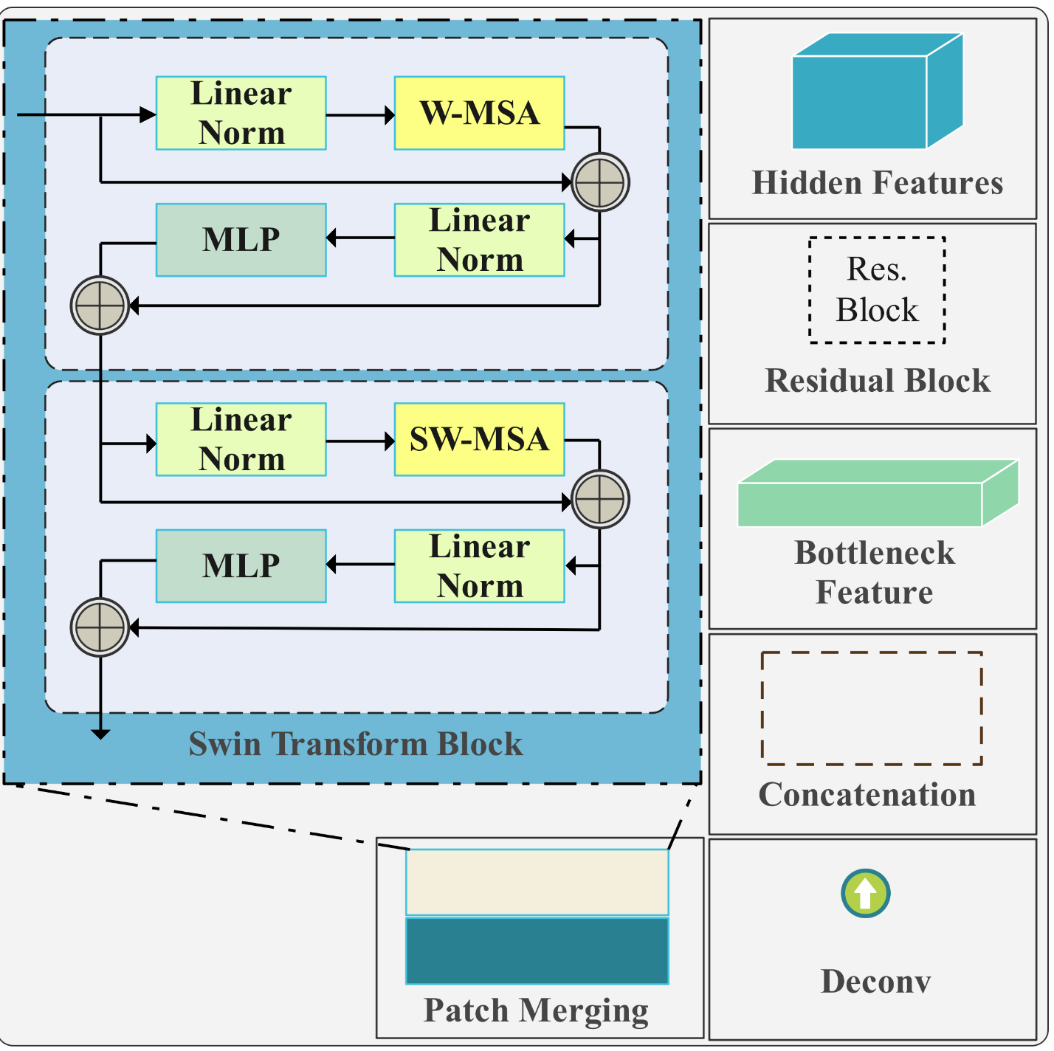
Fig. S1.** Network architecture guideline of Fig. 2. This figure shows components of Fig. 2 and the Swin Transformer block which consists of some parts including, Linear Norm (LN), window multi-head self-attention module (W-MSA), Multi-Layer Perceptron, and shifted window multi-head self-attention module (SW-MSA).

The Swin transformer block network can be detailed as follows:

The network initiates its process with the patch partitioning layer, which divides the input image into individual patches. Subsequently, the linear embedding layer reduces the dimension of the patches by projecting them into a C-dimensional space. The Swin Transformer Block consists of two main modules: the window multi-head self-attention module (W-MSA) and the shifted window multi-head self-attention module (SW-MSA). The input data first undergoes through the LayerNorm (LN) layer to normalize the activation values of the output from the previous layer and mitigate the problem of gradients vanishing. The output of LN is then directed into either the W-MSA layer or the SW-MSA layer. Compared to multi-headed self-attention (MSA), W-MSA offers significant computational savings by calculating each window independently.

While W-MSA reduces computation, it leads to limited information exchange between windows. To address this issue, SW-MSA is computed in subsequent blocks. Shifting the window down and right by half its size and recalculating W-MSA for the moved window facilitates information communication between windows. Consequently, the pairing of W-MSA and SW-MSA becomes necessary. After passing through either the W-MSA layer or the SW-MSA layer, the sequence proceeds through a batch normalization (BN) layer and concludes with a multilayer perceptron (MLP) for feature mapping, ultimately obtaining the final output. The Swin Transformer incorporates residual links to address the challenge of gradient vanishing. A patch merging layer is utilized to reduce the resolution by a factor of 2. Within this layer, the input undergoes splitting into regions of size 4 x 4 x 4, and voxels occupying identical positions within each region are merged to create a new patch. Linking these patches along the channel dimension alters the input image size from (W, H, D, C) to (W/2, H/2, D/2, 4C). Subsequently, a linear layer is employed to downsample the resolution by reducing the dimension to 2C.
